# Bis(benzimidazol-1-yl)methane dihydrate

**DOI:** 10.1107/S1600536811027966

**Published:** 2011-07-23

**Authors:** Yuping Fang, Shouwen Jin, Bingxia Chen, Yushuang Ge, Huabing Yin

**Affiliations:** a Tianmu College of ZheJiang A&F University, Lin’An 311300, People’s Republic of China

## Abstract

The bis­(benzimidazol-1-yl)methane mol­ecule of the title compound, C_15_H_12_N_4_·2H_2_O, displays a *trans* conformation with a twofold axis running through the methylene C atom. Two adjacent water mol­ecules are bonded to this mol­ecule through O—H⋯N hydrogen bonds, forming a trimer. Adjacent trimers are connected together *via* C—H⋯O inter­actions, forming a chain running along the *b*-axis direction. Two such chains are joined together *via* π–π inter­actions [centroid–centroid distance = 3.556 (2) Å], forming double chains, which are connected *via* the water mol­ecules through C—H⋯O associations, forming a sheet structure. The sheets are stacked on top of each other along the *a*-axis direction and connected through O—H⋯O and C—H⋯O inter­actions, forming a three-dimensional *ABAB* layer network structure.

## Related literature

For the use of bridged imidazole derivatives as multidentate N-donor ligands in the construction of functional coordination polymers, see: Chang *et al.* (2005 ▶); Wen *et al.* (2006 ▶); Fan *et al.* (2004 ▶); Abrahams *et al.* (2002 ▶); Jin *et al.* (2007 ▶); Ma *et al.* (2003 ▶). For the synthesis, see: Lavandera *et al.* (1988 ▶).
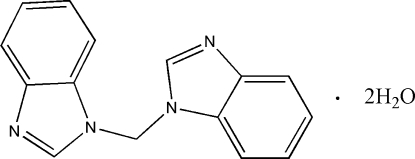


## Experimental

### 

#### Crystal data

C_15_H_12_N_4_·2H_2_O
*M* *_r_* = 284.32
Triclinic, 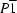

*a* = 8.3752 (9) Å
*b* = 9.2079 (8) Å
*c* = 10.7199 (10) Å
α = 100.288 (1)°
β = 101.495 (1)°
γ = 116.108 (2)°
*V* = 693.35 (12) Å^3^
*Z* = 2
Mo *K*α radiation
μ = 0.09 mm^−1^
*T* = 298 K
0.44 × 0.40 × 0.18 mm

#### Data collection

Bruker SMART CCD area-detector diffractometer
Absorption correction: multi-scan (*SADABS*; Bruker, 2002 ▶) *T*_min_ = 0.959, *T*_max_ = 0.983
3617 measured reflections
2411 independent reflections
1327 reflections with *I* > 2σ(*I*)
*R*_int_ = 0.021

#### Refinement

*R*[*F* ^2^ > 2σ(*F* ^2^)] = 0.050
*wR*(*F* ^2^) = 0.148
*S* = 1.01
2411 reflections
191 parameters
H-atom parameters constrained
Δρ_max_ = 0.18 e Å^−3^
Δρ_min_ = −0.18 e Å^−3^

### 

Data collection: *SMART* (Bruker, 2002 ▶); cell refinement: *SAINT* (Bruker, 2002 ▶); data reduction: *SAINT*; program(s) used to solve structure: *SHELXS97* (Sheldrick, 2008 ▶); program(s) used to refine structure: *SHELXL97* (Sheldrick, 2008 ▶); molecular graphics: *SHELXTL* (Sheldrick, 2008 ▶); software used to prepare material for publication: *SHELXTL*.

## Supplementary Material

Crystal structure: contains datablock(s) global, I. DOI: 10.1107/S1600536811027966/vm2104sup1.cif

Structure factors: contains datablock(s) I. DOI: 10.1107/S1600536811027966/vm2104Isup2.hkl

Supplementary material file. DOI: 10.1107/S1600536811027966/vm2104Isup3.cml

Additional supplementary materials:  crystallographic information; 3D view; checkCIF report

## Figures and Tables

**Table 1:** Hydrogen-bond geometry (Å, °)

*D*—H⋯*A*	*D*—H	H⋯*A*	*D*⋯*A*	*D*—H⋯*A*
O1—H1*E*⋯N2^i^	0.85	1.99	2.841 (3)	178
O1—H1*F*⋯O1^ii^	0.85	2.41	2.911 (5)	118
O2—H2*C*⋯N4^iii^	0.85	2.23	3.083 (3)	176
O2—H2*D*⋯N4^iv^	0.85	2.12	2.968 (3)	177
C2—H2⋯O1^ii^	0.93	2.39	3.299 (4)	167
C9—H9⋯O2^i^	0.93	2.56	3.363 (4)	145
C12—H12⋯O1^ii^	0.93	2.57	3.495 (4)	173

Symmetry codes: (i) 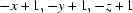; (ii) 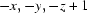; (iii) 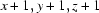; (iv) 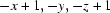.

**Fig. 1.**
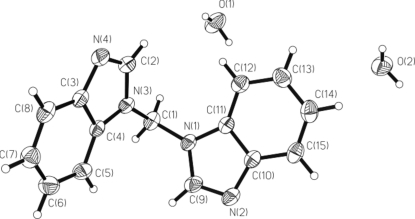
The structure of the title compound, showing the atom-numbering scheme. Displacement ellipsoids are drawn at the 30% probability level.

**Fig. 2.**
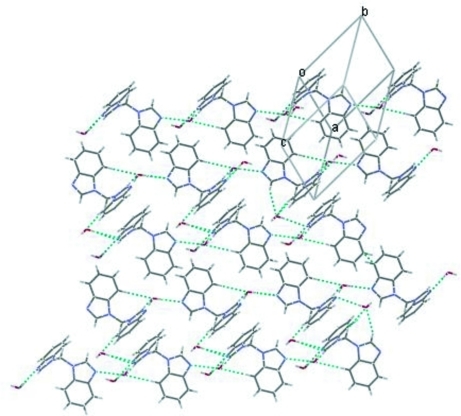
Two-dimensional sheet structure formed through hydrogen bonds (blue dashed lines) viewed along the a axis direction.

## supplementary crystallographic information

### Comment

Bridged imidazole derivatives can be used as multidentate N-donor ligands in constructing functioned coordination polymers, such as nonlinear optical materials (Chang *et al.*, 2005), novel hybrid inorganic organic photoactive materials (Wen *et al.*, 2006) and novel metal-organic frameworks (Fan *et al.*, 2004; Abrahams *et al.*, 2002). The ligands bearing alkyl spacers are a good choice of a N-donor ligand, and the flexible nature of the spacers allows the ligands to bend and rotate when coordinating to metal centers so as to conform to the coordination geometries of the metal ions. Significant progress has been achieved by us (Jin *et al.*, 2007) and others (Ma *et al.*, 2003) in this area.

However, the archived data on bridged benzimidazole derivatives bearing the methylene spacer have been rare. As an extension of our study in bridged imidazole derivatives, here in this paper, we report the structure of bis(benzimidazol-1-yl)methane dihydrate.

X-ray diffraction analysis indicated that in the title compound there are one bis(benzimidazol-1-yl)methane and two lattice water molecules (Fig. 1). All bond distances and angles are in the normal range. The r.m.s. deviation of the benzimidazole ring bearing the N1 and N2 atoms is 0.0056 Å. The r.m.s. deviation of the benzimidazole ring bearing the N3 and N4 atoms is 0.00123 Å. Both benzimidazole rings make a dihedral angle of 106.9 (3)° with each other. The bis(benzimidazol-1-yl)methane displays *trans* conformation with a twofold axis running through atom C1. Two water molecules are bonded to the bis(benzimidazol-1-yl)methane molecule through O—H···N hydrogen bonds (Table 1) to form an adduct. These adjacent adducts are connected together *via* C—H···O interactions to form a one-dimensional chain running along the *b* axis direction. There are two kinds of C—H···O associations (Table 1), one is arising from the N—CH—N of the benzimidazole moiety, another from the benzene C12—H12. In this chain the bis(benzimidazol-1-yl)methane molecules are parallelly arranged. Two such chains were joined together *via* the π-π interactions to form a double chain structure (*Cg*(1)*···Cg*(2)^i^ distance = 3.556 (2) Å, *Cg*(1) is the centroid of ring N1,N2, C9-C11, *Cg*(2) is the centroid of ring C10-C15, symmetry operation: (i) 1 - *x*, 1 - *y*, 1 - *z*). The bis(benzimidazol-1-yl)methane molecules at these two chains are arranged antiparallel. The double chains were connected together *via* the water molecules through the C—H···O associations to form a two-dimensional sheet extending along the direction forming a dihedral angle of *ca* 60 ° with the *bc* plane (Fig. 2). Such sheets were further stacked along the *a* axis direction *via* the O—H···O (between two water molecules with O···O separations of 2.911 Å) and C—H···O interactions to form a three-dimensional ABAB layer network structure.

### Experimental

The starting material bis(benzimidazol-1-yl)methane was prepared according to the published procedure (Lavandera *et al.*, 1988). A solid of bis(benzimidazol-1-yl)methane (24.8 mg, 0.10 mmol) in 10 ml of 95 percent EtOH was stirred at room temperature to dissolve it, then the solution was filtered into a test tube. The solution was left standing at room temperature for several days, colorless block crystals were isolated after slow evaporation of the solution in air at ambient temperature. The crystals were collected and dried in air to give the title compound.

### Refinement

H atoms bonded to the O atoms were located in a difference Fourier map, the O—H distance was kept 0.85 Å and refined isotropically. Other H atoms were positioned geometrically with C—H = 0.93–0.97 Å, and constrained to ride on their parent atoms with *U*_iso_(H) = 1.2 *U*_eq_(C).

### Crystal data

**Table:**

C_15_H_12_N_4_·2H_2_O	*V* = 693.35 (12) Å^3^
*M**_r_* = 284.32	*Z* = 2
Triclinic, *P*1	*F*(000) = 300
Hall symbol: -P 1	*D*_x_ = 1.362 Mg m^−^^3^
*a* = 8.3752 (9) Å	Mo *K*α radiation, λ = 0.71073 Å
*b* = 9.2079 (8) Å	µ = 0.09 mm^−^^1^
*c* = 10.7199 (10) Å	*T* = 298 K
α = 100.288 (1)°	Block, colorless
β = 101.495 (1)°	0.44 × 0.40 × 0.18 mm
γ = 116.108 (2)°	

### Data collection

**Table:**

Bruker SMART CCD area-detector diffractometer	2411 independent reflections
Radiation source: fine-focus sealed tube	1327 reflections with *I* > 2σ(*I*)
graphite	*R*_int_ = 0.021
phi and ω scans	θ_max_ = 25.0°, θ_min_ = 2.6°
Absorption correction: multi-scan (*SADABS*; Bruker, 2002)	*h* = −9→9
*T*_min_ = 0.959, *T*_max_ = 0.983	*k* = −10→10
3617 measured reflections	*l* = −10→12

### Refinement

**Table:**

Refinement on *F*^2^	Secondary atom site location: difference Fourier map
Least-squares matrix: full	Hydrogen site location: inferred from neighbouring sites
*R*[*F*^2^ > 2σ(*F*^2^)] = 0.050	H-atom parameters constrained
*wR*(*F*^2^) = 0.148	*w* = 1/[σ^2^(*F*_o_^2^) + (0.0658*P*)^2^ + 0.0465*P*] where *P* = (*F*_o_^2^ + 2*F*_c_^2^)/3
*S* = 1.01	(Δ/σ)_max_ < 0.001
2411 reflections	Δρ_max_ = 0.18 e Å^−^^3^
191 parameters	Δρ_min_ = −0.18 e Å^−^^3^
0 restraints	Extinction correction: *SHELXL97* (Sheldrick, 2008), Fc^*^=kFc[1+0.001xFc^2^λ^3^/sin(2θ)]^-1/4^
Primary atom site location: structure-invariant direct methods	Extinction coefficient: 0.005 (4)

### Special details

**Table:**

Geometry. All e.s.d.'s (except the e.s.d. in the dihedral angle between two l.s. planes) are estimated using the full covariance matrix. The cell e.s.d.'s are taken into account individually in the estimation of e.s.d.'s in distances, angles and torsion angles; correlations between e.s.d.'s in cell parameters are only used when they are defined by crystal symmetry. An approximate (isotropic) treatment of cell e.s.d.'s is used for estimating e.s.d.'s involving l.s. planes.
Refinement. Refinement of *F*^2^ against ALL reflections. The weighted *R*-factor *wR* and goodness of fit *S* are based on *F*^2^, conventional *R*-factors *R* are based on *F*, with *F* set to zero for negative *F*^2^. The threshold expression of *F*^2^ > σ(*F*^2^) is used only for calculating *R*-factors(gt) *etc*. and is not relevant to the choice of reflections for refinement. *R*-factors based on *F*^2^ are statistically about twice as large as those based on *F*, and *R*- factors based on ALL data will be even larger.

### Fractional atomic coordinates and isotropic or equivalent isotropic displacement parameters (Å^2^)

**Table:**

	*x*	*y*	*z*	*U*_iso_*/*U*_eq_	
N1	0.4209 (3)	0.3324 (3)	0.2893 (2)	0.0459 (6)	
N2	0.7039 (3)	0.5622 (3)	0.3488 (3)	0.0575 (7)	
N3	0.2169 (3)	0.0593 (3)	0.1258 (2)	0.0451 (6)	
N4	0.1831 (3)	−0.2003 (3)	0.0636 (2)	0.0573 (7)	
O1	−0.0360 (3)	0.1203 (3)	0.5805 (2)	0.0937 (8)	
H1E	0.0616	0.2167	0.6024	0.112*	
H1F	−0.0050	0.0476	0.5969	0.112*	
O2	0.7756 (3)	0.5078 (3)	0.9768 (2)	0.0843 (8)	
H2C	0.8861	0.5915	1.0009	0.101*	
H2D	0.7822	0.4171	0.9644	0.101*	
C1	0.2299 (4)	0.2083 (3)	0.2103 (3)	0.0511 (8)	
H1A	0.1780	0.2595	0.1549	0.061*	
H1B	0.1557	0.1745	0.2694	0.061*	
C2	0.1760 (4)	−0.0886 (4)	0.1540 (3)	0.0556 (8)	
H2	0.1458	−0.1089	0.2303	0.067*	
C3	0.2331 (4)	−0.1197 (3)	−0.0318 (3)	0.0454 (7)	
C4	0.2538 (3)	0.0428 (3)	0.0055 (3)	0.0409 (7)	
C5	0.2971 (4)	0.1488 (4)	−0.0736 (3)	0.0531 (8)	
H5	0.3109	0.2568	−0.0481	0.064*	
C6	0.3189 (4)	0.0870 (4)	−0.1916 (3)	0.0630 (9)	
H6	0.3456	0.1536	−0.2485	0.076*	
C7	0.3019 (4)	−0.0730 (4)	−0.2280 (3)	0.0610 (9)	
H7	0.3204	−0.1096	−0.3078	0.073*	
C8	0.2590 (4)	−0.1780 (4)	−0.1502 (3)	0.0547 (8)	
H8	0.2475	−0.2850	−0.1759	0.066*	
C9	0.5360 (4)	0.4721 (3)	0.2616 (3)	0.0553 (8)	
H9	0.4989	0.5015	0.1868	0.066*	
C10	0.6988 (4)	0.4744 (3)	0.4420 (3)	0.0457 (7)	
C11	0.5243 (4)	0.3304 (3)	0.4066 (3)	0.0431 (7)	
C12	0.4831 (4)	0.2208 (4)	0.4834 (3)	0.0533 (8)	
H12	0.3658	0.1250	0.4595	0.064*	
C13	0.6236 (5)	0.2602 (4)	0.5962 (3)	0.0643 (9)	
H13	0.6016	0.1883	0.6493	0.077*	
C14	0.7974 (4)	0.4042 (4)	0.6334 (3)	0.0658 (9)	
H14	0.8887	0.4272	0.7112	0.079*	
C15	0.8376 (4)	0.5132 (4)	0.5586 (3)	0.0571 (8)	
H15	0.9542	0.6104	0.5848	0.069*	

### Atomic displacement parameters (Å^2^)

**Table:**

	*U*^11^	*U*^22^	*U*^33^	*U*^12^	*U*^13^	*U*^23^
N1	0.0420 (13)	0.0347 (13)	0.0527 (15)	0.0176 (11)	0.0083 (12)	0.0057 (11)
N2	0.0515 (16)	0.0387 (14)	0.0686 (17)	0.0166 (12)	0.0115 (14)	0.0092 (13)
N3	0.0420 (13)	0.0337 (13)	0.0507 (14)	0.0156 (10)	0.0099 (11)	0.0066 (11)
N4	0.0621 (16)	0.0388 (14)	0.0582 (16)	0.0194 (12)	0.0095 (13)	0.0112 (13)
O1	0.0680 (15)	0.0786 (17)	0.1061 (19)	0.0124 (12)	0.0326 (14)	0.0224 (15)
O2	0.0832 (17)	0.0511 (14)	0.112 (2)	0.0364 (12)	0.0122 (15)	0.0216 (13)
C1	0.0415 (16)	0.0475 (17)	0.0584 (19)	0.0239 (14)	0.0094 (14)	0.0044 (15)
C2	0.0492 (18)	0.0466 (18)	0.0557 (19)	0.0133 (14)	0.0107 (15)	0.0159 (16)
C3	0.0386 (15)	0.0358 (15)	0.0510 (18)	0.0164 (12)	0.0035 (13)	0.0060 (14)
C4	0.0328 (14)	0.0348 (15)	0.0471 (17)	0.0155 (12)	0.0047 (13)	0.0068 (13)
C5	0.0533 (18)	0.0411 (17)	0.066 (2)	0.0240 (14)	0.0182 (16)	0.0187 (16)
C6	0.064 (2)	0.061 (2)	0.065 (2)	0.0281 (17)	0.0258 (17)	0.0230 (17)
C7	0.060 (2)	0.062 (2)	0.057 (2)	0.0295 (17)	0.0209 (16)	0.0093 (17)
C8	0.0478 (17)	0.0402 (17)	0.063 (2)	0.0208 (14)	0.0067 (15)	0.0011 (15)
C9	0.062 (2)	0.0343 (16)	0.064 (2)	0.0220 (15)	0.0160 (17)	0.0118 (15)
C10	0.0442 (16)	0.0380 (16)	0.0522 (18)	0.0220 (14)	0.0138 (14)	0.0040 (14)
C11	0.0411 (16)	0.0410 (16)	0.0460 (17)	0.0220 (13)	0.0142 (13)	0.0049 (13)
C12	0.0458 (18)	0.0543 (19)	0.058 (2)	0.0212 (15)	0.0230 (16)	0.0138 (16)
C13	0.066 (2)	0.077 (2)	0.056 (2)	0.0363 (19)	0.0247 (18)	0.0244 (18)
C14	0.056 (2)	0.084 (3)	0.055 (2)	0.037 (2)	0.0141 (17)	0.0121 (19)
C15	0.0462 (18)	0.056 (2)	0.057 (2)	0.0235 (15)	0.0120 (16)	−0.0021 (16)

### Geometric parameters (Å, °)

**Table:**

N1—C9	1.353 (3)	C4—C5	1.382 (4)
N1—C11	1.385 (3)	C5—C6	1.373 (4)
N1—C1	1.445 (3)	C5—H5	0.9300
N2—C9	1.308 (3)	C6—C7	1.389 (4)
N2—C10	1.389 (3)	C6—H6	0.9300
N3—C2	1.356 (3)	C7—C8	1.364 (4)
N3—C4	1.383 (3)	C7—H7	0.9300
N3—C1	1.450 (3)	C8—H8	0.9300
N4—C2	1.312 (4)	C9—H9	0.9300
N4—C3	1.393 (3)	C10—C11	1.390 (4)
O1—H1E	0.8499	C10—C15	1.392 (4)
O1—H1F	0.8500	C11—C12	1.386 (4)
O2—H2C	0.8500	C12—C13	1.371 (4)
O2—H2D	0.8500	C12—H12	0.9300
C1—H1A	0.9700	C13—C14	1.384 (4)
C1—H1B	0.9700	C13—H13	0.9300
C2—H2	0.9300	C14—C15	1.364 (4)
C3—C8	1.384 (4)	C14—H14	0.9300
C3—C4	1.400 (3)	C15—H15	0.9300
			
C9—N1—C11	106.2 (2)	C5—C6—H6	119.3
C9—N1—C1	126.7 (2)	C7—C6—H6	119.3
C11—N1—C1	127.1 (2)	C8—C7—C6	122.0 (3)
C9—N2—C10	103.8 (2)	C8—C7—H7	119.0
C2—N3—C4	106.7 (2)	C6—C7—H7	119.0
C2—N3—C1	126.1 (2)	C7—C8—C3	117.7 (3)
C4—N3—C1	127.1 (2)	C7—C8—H8	121.2
C2—N4—C3	104.4 (2)	C3—C8—H8	121.2
H1E—O1—H1F	109.6	N2—C9—N1	114.4 (3)
H2C—O2—H2D	108.3	N2—C9—H9	122.8
N1—C1—N3	112.1 (2)	N1—C9—H9	122.8
N1—C1—H1A	109.2	N2—C10—C11	110.6 (2)
N3—C1—H1A	109.2	N2—C10—C15	129.4 (3)
N1—C1—H1B	109.2	C11—C10—C15	120.0 (3)
N3—C1—H1B	109.2	N1—C11—C12	133.1 (2)
H1A—C1—H1B	107.9	N1—C11—C10	105.0 (2)
N4—C2—N3	113.8 (3)	C12—C11—C10	122.0 (3)
N4—C2—H2	123.1	C13—C12—C11	116.8 (3)
N3—C2—H2	123.1	C13—C12—H12	121.6
C8—C3—N4	130.0 (3)	C11—C12—H12	121.6
C8—C3—C4	120.0 (3)	C12—C13—C14	121.8 (3)
N4—C3—C4	109.9 (2)	C12—C13—H13	119.1
C5—C4—N3	132.7 (2)	C14—C13—H13	119.1
C5—C4—C3	122.1 (3)	C15—C14—C13	121.5 (3)
N3—C4—C3	105.1 (2)	C15—C14—H14	119.3
C6—C5—C4	116.7 (3)	C13—C14—H14	119.3
C6—C5—H5	121.7	C14—C15—C10	117.9 (3)
C4—C5—H5	121.7	C14—C15—H15	121.0
C5—C6—C7	121.4 (3)	C10—C15—H15	121.0
			
C9—N1—C1—N3	98.4 (3)	N4—C3—C8—C7	177.7 (3)
C11—N1—C1—N3	−80.0 (3)	C4—C3—C8—C7	−0.9 (4)
C2—N3—C1—N1	96.9 (3)	C10—N2—C9—N1	0.0 (3)
C4—N3—C1—N1	−79.1 (3)	C11—N1—C9—N2	−0.2 (3)
C3—N4—C2—N3	0.1 (3)	C1—N1—C9—N2	−178.9 (2)
C4—N3—C2—N4	0.2 (3)	C9—N2—C10—C11	0.3 (3)
C1—N3—C2—N4	−176.5 (2)	C9—N2—C10—C15	−178.7 (3)
C2—N4—C3—C8	−179.1 (3)	C9—N1—C11—C12	179.9 (3)
C2—N4—C3—C4	−0.4 (3)	C1—N1—C11—C12	−1.5 (4)
C2—N3—C4—C5	177.7 (3)	C9—N1—C11—C10	0.4 (3)
C1—N3—C4—C5	−5.6 (4)	C1—N1—C11—C10	179.1 (2)
C2—N3—C4—C3	−0.4 (3)	N2—C10—C11—N1	−0.5 (3)
C1—N3—C4—C3	176.2 (2)	C15—C10—C11—N1	178.6 (2)
C8—C3—C4—C5	1.0 (4)	N2—C10—C11—C12	180.0 (2)
N4—C3—C4—C5	−177.9 (2)	C15—C10—C11—C12	−0.9 (4)
C8—C3—C4—N3	179.4 (2)	N1—C11—C12—C13	−179.8 (3)
N4—C3—C4—N3	0.5 (3)	C10—C11—C12—C13	−0.4 (4)
N3—C4—C5—C6	−177.8 (3)	C11—C12—C13—C14	1.2 (4)
C3—C4—C5—C6	0.1 (4)	C12—C13—C14—C15	−0.6 (5)
C4—C5—C6—C7	−1.3 (4)	C13—C14—C15—C10	−0.8 (4)
C5—C6—C7—C8	1.4 (5)	N2—C10—C15—C14	−179.6 (3)
C6—C7—C8—C3	−0.3 (4)	C11—C10—C15—C14	1.5 (4)

### Hydrogen-bond geometry (Å, °)

**Table:**

*D*—H···*A*	*D*—H	H···*A*	*D*···*A*	*D*—H···*A*
O1—H1E···N2^i^	0.85	1.99	2.841 (3)	178.
O1—H1F···O1^ii^	0.85	2.41	2.911 (5)	118.
O2—H2C···N4^iii^	0.85	2.23	3.083 (3)	176.
O2—H2D···N4^iv^	0.85	2.12	2.968 (3)	177.
C2—H2···O1^ii^	0.93	2.39	3.299 (4)	167
C9—H9···O2^i^	0.93	2.56	3.363 (4)	145
C12—H12···O1^ii^	0.93	2.57	3.495 (4)	173

Symmetry codes: (i) −*x*+1, −*y*+1, −*z*+1; (ii) −*x*, −*y*, −*z*+1; (iii) *x*+1, *y*+1, *z*+1; (iv) −*x*+1, −*y*, −*z*+1.

## References

[bb1] Abrahams, B. F., Hoskins, B. F., Robson, R. & Slizys, D. A. (2002). *CrystEngComm*, **4**, 478–482.

[bb2] Bruker (2002). *SADABS*, *SMART* and *SAINT* Bruker AXS Inc., Madison, Wisconsin, USA.

[bb3] Chang, Q., Meng, X. R., Song, Y. L. & Hou, H. W. (2005). *Inorg. Chim. Acta*, **358**, 2117–2124.

[bb4] Fan, J., Sun, W. Y., Okamura, T., Zheng, Y. Q., Sui, B., Tang, W. X. & Ueyama, N. (2004). *Cryst. Growth Des.* **4**, 579–584.

[bb5] Jin, S. W. & Chen, W. Z. (2007). *Inorg. Chim. Acta*, **12**, 3756–3764.

[bb6] Lavandera, J. L., Cabildo, P. & Claramunt, R. M. (1988). *J. Heterocycl. Chem.* **25**, 771–778.

[bb7] Ma, J. F., Yang, J., Zheng, G. L., Li, L. & Liu, J. F. (2003). *Inorg. Chem.* **42**, 7531–7534. 10.1021/ic034846q 14606848

[bb8] Sheldrick, G. M. (2008). *Acta Cryst.* A**64**, 112–122. 10.1107/S0108767307043930 18156677

[bb9] Wen, L. L., Li, Y. Z., Lu, Z. D., Lin, J. G., Duan, C. Y. & Meng, Q. J. (2006). *Cryst. Growth Des.* **6**, 530–537.

